# Developing the SCM workforce in Nigeria through contextualised pre-service education and continued professional development

**DOI:** 10.1186/2052-3211-7-S1-O22

**Published:** 2014-12-17

**Authors:** Adebayo Adekola, Adenike Adelanwa

**Affiliations:** 1 Supply Chain Management Systems (SCMS), Abuja, Nigeria

## Background

The human resource crisis extends into all areas of a health system—including the supply chain. Access to quality HIV commodities for testing, treatment and care can be impeded by staff lacking skills in health supply chain management (SCM) often resulting in stock-outs and expiries. In Nigeria, SCMS set out to build in-country capacity to accelerate current and future health workforce education in SCM through three distinct learning modalities.

## Method

SCMS implemented a three-pronged approach to SCM education through pre-service, in-service and e-learning training by engaging: the incoming supply chain workforce by working with 12 pharmacy schools, health personnel by working with the Institute of Public Health at Obafemi Awolowo University Ife to implement a logistics management of health commodities course, and with the growing need for laboratory logistics skills by working with the K4Health project and two credentialing bodies to develop the SCM content.

## Results

This approach has built the capacity of more than 30 instructors at 13 academic and training institutions reaching over 2,300 students with ongoing expansion to 20 schools (both public and private) with medical laboratory science undergraduate programme (BMLS), and eight state schools of health technology. Pre-service training in supply chain management has seen close to 400 pharmacy students graduate with this knowledge, as of March 2014. The Institute of Public Health, Obafemi Awolowo University has also completed three training rounds of the logistics management of health commodities course with a total attendance of 52 health personnel drawn from public and private organizations.

## Discussion

The Nigerian health workforce gained critical SCM skills to ensure continued patient access to life-saving medicines. These modalities present a sustainable capacity building model given their full adoption by local institutions and faculty. This approach can be applied to other knowledge areas critical to the HIV workforce further enhancing country ownership. With low start-up and maintenance costs, this three-pronged effort has proven potential to save thousands of dollars by reducing dependency on costly in-service training.

## Lessons learned

The program’s success is due to stakeholder engagement and buy-in, strategic use of existing educational structures, professional bodies and MOH’s commitment. In-service training for 30 participants ranges from US$31,000-$50,000 which must be repeated over time while pre service training and e-learning require one-time costs for initial implementation with minimal continuous costs.

